# Development of the “living well” concept for older people with dementia

**DOI:** 10.1186/s12877-023-04304-3

**Published:** 2023-09-29

**Authors:** Jiyoung Kim, Nayeon Shin

**Affiliations:** 1 Department of Nursing, Inha University, 100 Inha-ro, Michuhol-gu, Incheon, 22212 Republic of Korea; 2 Unit manager, CHA Bundang Medical Center, CHA University, Seongnam, Republic of Korea

**Keywords:** Concept analysis, Older people, Dementia, Living well

## Abstract

**Background:**

An important goal in dementia care is how people with dementia can be supported in living well. To this end, we need a conceptualization of “living well” that is suitable for older people with dementia and then develop this conceptualization from the perspectives of both older people and caregivers. This study analyzed the concept of “living well” among older people with dementia.

**Methods:**

Following Schwartz-Barcott and Kim’s hybrid model, this study comprised theoretical, fieldwork, and final analytic phases. In the theoretical phase, we reviewed the extant literature. In the fieldwork phase, we conducted in-depth interviews with 12 participants (five older people with dementia, two family members, and five nurses), followed by qualitative content analysis. In the final analytic phase, we defined the concept of “living well” by comprehensively analyzing the data from the theoretical phase and results from the fieldwork phase.

**Results:**

We derived physical, mental, and social relationship dimensions of the conceptualization of “living well” for older people with dementia. The physical dimensions were “ability for daily living” and “symptom management.” The mental dimensions were “psychological health,” “psychological stability,” “maintaining identity and growth,” and “human dignity.” Finally, the social relationship dimensions were “maintaining social relations and community connectivity” and “government support.”

**Conclusions:**

Our analysis of the concept of “living well” can be used for developing tools and interventions to improve the ability of older people with dementia to live well.

## Introduction

Globally, dementia is a major cause of disability and dependency among older people and is the seventh most common cause of death. As of 2023, approximately 55 million people have dementia worldwide, and this number is projected to increase by 10 million annually. Moreover, dementia has substantial social costs. Globally, dementia costed economies 1.3 trillion USD in 2019. Approximately half of these expenses were related to informal caregivers (e.g., family members and close friends), who devote an average of five hours per day to care and supervision [1]. Further, South Korea (hereafter, Korea) has a high proportion of older people 65 years or older with dementia at approximately 830,000 (10.2%) of the 8.13 million older people. Moreover, the social and economic burden caused by dementia is steadily rising. In 2020, approximately 365,000 people with dementia used long-term care insurance in Korea, and the total cost of care was approximately KRW 4.9 trillion (3.7 billion USD). The national dementia management cost for people with dementia was KRW 17.3 trillion for the same year (13 billion USD), or approximately 0.9% of the gross domestic product; this cost is expected to rise to approximately KRW 56.9 trillion (43 billion USD) by 2040 [2].

Over the past decade, dementia policies worldwide have shifted from focusing on the negative consequences of disability to positive ones, including the perception that it is a manageable condition provided there is appropriate support [3]. For instance, the 2017–2025 Global Action Plan on the Public Health Response to Dementia aims to improve the lives of people with dementia and their caregivers while reducing the impact of dementia on communities and countries [4]. Korea is also establishing a comprehensive dementia management plan to reduce the burden on individuals and society and develop appropriate dementia management measures. The 4th National Dementia Plan (2021–2025) seeks to establish a society that ensures safety for people with dementia by bringing them, their families, and local communities together [5]. It establishes core policies and support services to improve the daily lives of consumers, people with dementia, families, and providers.

Importantly, in the management and treatment of people with chronic diseases and disabilities, the focus is not on “living longer” but on “living well” while maintaining the quality of daily life [6]. Dementia is untreatable, and people with dementia gradually lose cognitive function [7], with an average life expectancy of 3–8 years after diagnosis [8]. Thus, optimizing the ability of people with dementia to live well and understanding how to support them in this endeavor are important goals [9, 10]. A UK-based longitudinal cohort study of older people with dementia and family caregivers found that living well is a broader and more multifaceted concept than quality of life. Specifically, living well integrates the concepts of well-being and life satisfaction and reflects social capital, assets and resources, and social participation [11, 12]. Studies in Korea have explored the factors affecting the quality of life of older people with dementia [13]. For instance, one study examined the effect of behavioral and psychological symptom relief programs for older people with dementia on their quality of life [14]. However, the concept of living well has not been clearly defined, and limitations exist in explaining the existing concept of quality of life. Therefore, further research is needed on comprehensive conceptualizations of living well to adequately support the healthcare community.

Living well is ideally defined by the patient’s values and goals regarding physical, mental, and social functioning. However, conceptualizing and evaluating living well is challenging because patient perspectives vary [3, 15]. In particular, the viewpoints and needs of study participants and their family caregivers should be considered to understand the meaning of living well for older people with dementia [3]. Dementia research in Korea has highlighted the problem of ignoring individuality and diversity as well as excluding the viewpoints and experiences of patients [13, 16] and standardizing dementia symptoms [16]. As limitations exist in generalizing the concept from the social and cultural contexts social and cultural contexts from studies conducted outside Korea, we need to conceptualize living well in a manner that is suitable for the older people with dementia in Korea, and develop a conceptualization that considers the perspectives of both older people with dementia and their caregiver(s).

This study aimed to develop the concept of living well for older people with dementia in Korea by embracing their perspectives and that of their caregivers (family members and nurses) using Schwartz-Barcott and Kim’s hybrid model [17]. Developing a comprehensive concept of living well for older people with dementia can provide an appropriate basis for establishing theory and developing robust interventions for their care.

## Methods

### Study design

To analyze the concept of “living well” for older people with dementia, this study adopted Schwartz-Barcott and Kim’s hybrid model, and comprised three phases—namely, theoretical, fieldwork, and final analytic [17]. Among the various methods of analyzing concepts in nursing, Schwartz-Barcott and Kim’s [17] hybrid model is based on perceptions formed in the field and via a literature review. The characteristics of older people with dementia should be accurately reflected to establish a comprehensive concept of living well. Thus, a hybrid model with theoretical analysis and fieldwork is appropriate for exploring the meaningful conceptual phenomena of interest.

### Procedure

#### Theoretical phase

In the theoretical phase, the literature published in Korea and abroad until February 2022 was searched using the Cumulative Index to Nursing and Allied Health Literature (CINAHL), PubMed, ProQuest, Research Information Sharing Service (RISS), the Korean Studies Information Service System (KISS), DBpia, and Google Scholar. The search terms included dementia, Alzheimer’s disease, caregiver, family, and well-being status. Overall, 29 articles were extracted after removing redundant data, reviewing the titles and abstracts, and confirming whether they adequately addressed the topic of living well for older people with dementia. The inclusion criteria were as follows: studies that (1) contained elements suitable for understanding the meaning of living well in older people with dementia; and (2) included the viewpoints of older people with dementia, family caregivers, and healthcare professionals. The exclusion criteria were as follows: studies that (1) included only the views of family caregivers and healthcare professionals and (2) were not written in Korean or English.

#### Fieldwork phase

In the fieldwork phase, the concepts analyzed in the theoretical phase were empirically validated through qualitative research methods. Specifically, in-depth interviews were conducted with older people with dementia and their caregivers; the interview data were subsequently analyzed using qualitative content analysis.

### Setting and sample

The interview participants included older people with dementia and their caregivers who could express themselves and communicate with others. Older people with dementia were defined as those aged 65 years or older diagnosed with mild cognitive impairment or mild-to-moderate dementia by a neurologist. The criteria for selecting caregivers were family members and nurses with more than one year of direct care experience for older people with mild cognitive impairment and dementia. Through theoretical purposive sampling, we selected participants who provided abundant information on the living conditions of older people with dementia.

### Data collection

Older people with dementia may experience various degrees of living well depending on the severity of dementia, their family situation, living environment, and socio-economic status. Considering this, we employed the snowball sampling method [18]. Specifically, participants who fulfilled the selection criteria were introduced through doctors and head nurses in medical institutions; subsequently, these participants introduced other potential participants. Table 1 lists the participants’ characteristics.

**Table 1:** Participant characteristics in the fieldwork phase

Older people with dementia (n = 5)	Gender	Age (yrs)	Diagnosis	Year of diagnosis	Family	Living environment	Socio-economic status
Older people 1	Female	66	Alzheimer’s disease (moderate), HTN	2017	Spouse, 2 sons	Living at home, attending a welfare center	Self-employed
Older people 2	Male	78	Alzheimer’s disease (moderate), HTN, DM, Asthma	2014	Spouse, 2 daughters, 1 son	Living at home, attending a day care center	Unemployed
Older people 3	Female	66	Mild cognitive impairment	2019	1 son, 1 daughter	Living at home, under cognitive rehabilitation	Housewife, former mart worker
Older people 4	Male	68	Alzheimer’s disease (mild)	2019	Spouse, 2 daughters	Living at home, under cognitive rehabilitation	Unemployed, former teacher
Older people 5	Female	70	Mild cognitive impairment	2018	3 sons	Living at home, under cognitive rehabilitation	Artist
**Family caregivers (n = 2)**	**Gender**	**Age** **(yrs)**	**Diagnosis**	**Year of diagnosis**	**Family**	**Living environment**	**Socio-economic status**
Family 1	Female	67	Acholic dementia (mild)	2019	Spouse (Older people), daughter	Living at home, attending a day care center	Homemaker
Family 2	Female	70	Alzheimer’s disease (moderate), HTN DM	2017	Spouse (Older people), 2 daughters	Living at home, daughter lives nearby	Homemaker
**Professional caregivers (n = 5)**	**Gender**	**Age** **(yrs)**	**Working department**	**Current department working period (yr)**	**Education**	**Position**	**Patients primarily cared for**
Nurse 1	Female	45	Rehabilitation ward	5	Bachelors	Head nurse	MCI
Nurse 2	Female	65	Nursing hospital	3	Masters	Director	Dementia
Nurse 3	Female	44	Geriatric hospital	8	Bachelors	Charge nurse	MCI, dementia
Nurse 4	Female	50	Nursing hospital	11	Bachelors	Head nurse	Mild cognitive impairment
Nurse 5	Female	49	Neurology ward	16	Bachelors	Head nurse	MCI

HTN: Hypertension; DM: Diabetes mellitus; MCI: Mild cognitive impairment

The researcher prepared interview questions based on the literature and had them reviewed by a nursing professor with extensive experience in qualitative research. The interview questions for older people with dementia were as follows: “What do you think life should be like for older people with dementia?” and “What do you think is the meaning of living well?” The questions for caregivers were as follows: (1) “Tell me about your overall impression of the daily life of older people with dementia,” (2) “What do you think living well (living like a human, meaningfully, and properly) looks like?”, (3) “What do you think living well is for older people with dementia with respect to their progress and treatment status?,” and (4) “What do you think living well is for older people with dementia considering their family and socio-economic circumstances?” We conducted semi-structured interviews, recorded them after obtaining participants’ consent, and transcribed them immediately afterwards. The researcher also closely observed the participants’ words, behaviors, facial expressions, tone of voice, eye movements, emotions, clothing, and tone of voice during the interviews.

Data were collected from August 2019 to July 2020. The interviews lasted from 30 min to 1 h, and follow-up interviews were conducted when additional questions arose after analysis. Overall, in-depth interviews were conducted with five older people with dementia, two family members, and five nurses until we reached saturation and no new ideas or opinions emerged.

### Data analysis

Data were analyzed using the qualitative content analysis process in three main phases: preparation, organizing, and reporting [19]. Based on the theoretical phase’s results, a deductive approach was adopted to verify and confirm them in the fieldwork phase. In the preparation phase of data analysis, meaningful words were identified and coded using words as the unit of analysis. Second, in the organizing phase, the qualitative data were checked for compatibility with the physical, mental, and social relationship dimensions of the theoretical phase, and coded for agreement or to serve as an example. Open coding was conducted for data that did not fit the theoretical phase. Specially, notes and headings were written in the text while reading it according to the inductive content analysis, and then, themes were derived. In the final reporting phase, the results were derived through final analysis and confirmed.

### Final analytic phase

In the final analytic phase, the theoretical phase data (literature review) and fieldwork results were comprehensively analyzed through a discussion process to identify the attributes, antecedents, and consequences of living well for older people with dementia. An additional review was requested from a nursing professor with extensive experience in qualitative research, who verified the validity of the anaysis, and a final agreement was reached.

### Ethical considerations

This study was approved by the Institutional Review Board (IRB) of the Researchers’ University (IRB approval number: DIRB-201,906-HR-E-14). The researcher explained the purpose of the study, progress of the interview, management and disposal of the collected data, and participation in and discontinuation of the interviews. As the study included older people with dementia who were vulnerable, sufficient time was devoted to the recruitment process and explanation. Before conducting the interviews, written informed consent was obtained from each participant after clarifying the study’s aims and procedures.

## Results

### Theoretical phase

#### Characteristics and definition of the concept

According to Collins’ Essential English Dictionary [20], living well can be interpreted as “living luxuriously,” “living a moral life,” and “living comfortably.” In the YBM dictionary, living well is described as “living well [without inconvenience]” and “living a moral life” [21]. In summary, these two dictionaries define living well as having a comfortable and moral life, beyond simply being materially affluent.

In the context of chronic diseases, living well is defined as the optimal achievable state of health that encompasses all dimensions of physical, mental, and social well-being and is characterized by self-perceived comfort, function, and life satisfaction [15]. For older people with dementia, living well is generally identified as an important indicator of their quality of life and the treatment they recieve. However, living well can also mean a higher quality of life at a specific point in time [22]. Studies have emphasized that the experience of dementia should not be considered an isolated and abstract phenomenon. The relational context in which personality is realized, the formation of interactions, and the support provided within the relationship are also important [16, 23]. Therefore, for older people with dementia, living well can be a concept that encompasses the physical, mental, and social dimensions.

### Attributes

We derived specific attributes of the concept of living for older people with dementia in relation to the physical, mental, and social relationship dimensions (see Table 2).

**Table 2:** Dimension, attributes, and themes of the concept of “living well” for older people with dementia in the fieldwork phase

Dimension	Attributes	Themes	Quote
Physical	Ability for daily living	• Maintaining independence in daily living • Setting personal goals for daily living	• I quit my job and kept forgetting things. I have a poor memory; thus, my self-confidence is low, and I always get depressed about everything. (Older people 3) • If my daily life is ruined, I cannot live well. I can live well only if I can perform daily activities (Older people 5) • The things that older people with dementia want to do are highly routine-specific, such as cooking for their grandchildren and taking them to and from school. (Nurse 1)
	Symptom management	• Cognitive enhancement • Behavioral and psychological symptoms management • Physical disease and symptom management	• I am trying to participate in cognitive rehabilitation therapy even if it means I have less time for myself. (Older people 5) • Since these patients need to solve cognitive and physical problems, it is necessary to grasp the cognitive, physical and holistic aspects… (Nurse 2) • Since cognitive function declines, language function deteriorates, making daily life ability challenging. Paying attention and efforts to prevent further progression will be important in determining whether they will live well. (Nurse 5)
Mental	Psychological health	• Self-efficacy • Hope • Willingness to live well	• People in their 60s are not grandmothers these days, right? I am still in my prime… (Older people 3) • The fact that I am willing to live well right now proves that I am living well. (Older people 4) • I do not think older people with dementia are cognitively or emotionally deteriorated. We need to instill even more hope. (Nurse 4)
	Psychological stability	• Normality • Comfort	• Eat well and live well. (Older people 2) • I hope my husband’s mental condition does not change, and I want to be comfortable when I wake up. (Family 2) • I think the patient is living a comfortable life. (Nurse 3)
	Maintaining identity	• Sense of self • Self-esteem • Positive life review	• I think it was a life that was recognized and rewarded by many people… (Omitted) In the future, I want to be recognized by others for the rest of my life while expressing and leaving behind more work. (Older people 5) • Rehabilitation is like finding a forgotten piece of memory again. It is good that memories buried deep in your head can be recalled, and you are happy and think of other memories while thinking about them at home. Recalling the happy memories of the past and living happily every day… (Older people 6)
	Human dignity	• Meeting basic needs • Maintaining dignity	• They say that people have to maintain dignity until they die, which is what makes them blessed. (Older people 3) • If the basics do not work, the complex ones will not work. (Nurse 2)
Social relationship	Maintaining social relations and community connectivity	• Main caregiver support • Social engagement and relationship building	• I become more depressed when I am alone. (Older people 3) • I will meet familiar people around me to live well… (Older people 4) • I think it would be nice if he met other people too. (Family 1) • I wonder if having someone next to me who can accept me like a wife or child is living a good life. (Family 2) • It is important to make the relationship of these people with whom… (Omitted) Making them feel like they have someone they can care for, communicate with, and maintain a relationship with. (Nurse 5)
	Government support	• Facilities for older people with dementia • Financial support • Professional workforce support	• I wish other people would help me so I do not have to be a burden on my children. Why not send some people from the district office and provide free medicine at the public health center? (Older people 3) • The patient still goes to the daycare center in the morning. I want the country to look at the citizen without any burden. (Family 1) • I think the welfare system for the socially underprivileged should be expanded so that they do not have to worry about finances. (Nurse 1)

Regarding the physical dimension, the first attribute was having the “ability for daily living” [15, 23–26]. Older people with dementia with functional disability may have a lower ability to live well, as even slight differences in functional ability can have a negative impact [25]. Some researchers have suggested that supporting the functional ability and independence of older people with dementia is crucial [24]. The second attribute was “symptom management.” Dementia-related [12, 22, 25, 27] and psychological symptoms (e.g., delusions and hallucinations) [28] significantly impact older people’s life experiences and quality of life. Therefore, dementia-related symptoms, defects, and disabilities must be managed appropriately [3, 6].

Next, the first attribute of the mental dimension was “psychological health.” Good psychological health has both direct and indirect effects on living well [3, 6, 22, 28–31]. The second was “psychological stability.” In one study, older people with dementia enjoyed old age, were satisfied with their current life, and tried experiencing and maintaining the continuity of life before and after diagnosis [31]. The third attribute was “maintaining identity and growth.” Studies have revealed that older people with dementia acknowledge a continuous sense of self and meaning even after diagnosis and grow while maintaining their identity through positive perspectives on life [31]. One study suggested that older people with dementia actively preserve their identities, which allows them to re-evaluate their self-understanding and meaning, leading to their growth as human beings [31].

Finally, the first attribute of the social relationship dimension was “maintaining social relations and community connectivity” [3, 12, 22, 29–34]. Positive relationships include social contact and amicable relationships with others. Social connectivity and promoting bonding are essential for addressing the negative effects associated with social isolation [32, 34]. Older people with dementia who live alone or have no caregivers are a vulnerable group at a high risk of their social, psychological, environmental, and medical needs not being fulfilled [35]. Older people with dementia without a caregiver are less able to live well than those with a caregiver [36]. The second attribute was “government support.” Whether older people with dementia will live well is a matter of social justice. Accordingly, governments should improve welfare policies [32] and provide professional workforce to support them [7, 22, 23].

### Antecedents

The first antecedent for living well for older people with dementia was utilizing a “person-centered approach.” Studies have suggested that a person-centered approach is important for prioritizing the intrinsic values of older people with dementia, defending their rights, and enhancing their abilities [16, 33, 36–39].

The second antecedent was the “perception of dementia.” Interventions and services for living well should consider the perceptions of older people with dementia [10, 40], caregivers [41], and healthcare professionals [26, 41]. Appropriate treatment should be provided by understanding aging, dementia status, and awareness of difficulties or changes related to the diagnosis in older people with dementia [10, 40]. Moreover, caregivers’ stress levels, perceived social restrictions, and caregiving competence can affect the ability of older people with dementia to live well and their care experience [41]. Further, healthcare professionals need to acknowledge that older people with dementia can live well with their condition and enjoy a better life; accordingly, professionals should appropriately guide older people with dementia and caregivers to available facilities and services [26, 41].

The third antecedent was “shared decision-making.” Living with dementia involves losing one’s independence and seeking support from others in all aspects of life. Therefore, shared decision-making and cooperative efforts are essential for older people with dementia to live well [7, 26]. Caring for them involves rebuilding relationships through shared decision-making and collaboration, especially deciding how to live well with dementia. To help them live well, the needs of older people with dementia should be identified, and autonomy and decision-making authority should be granted through discussions involving the family. During the shared decision-making process, cooperation with family members and acquaintances who have maintained close relationships with older people with dementia is recommended as communication may be difficult owing to their declining communication skills and independence [7].

### Consequences

The literature review revealed that in the long term, living well improves the psychological adaptation and adaptation to disease of older people with dementia [29, 42, 43].

### Fieldwork phase

#### Attributes

In the fieldwork phase, we derived several attributes in the physical, mental, and social relationship dimensions of living well for older people with dementia. By analyzing the fieldwork data, we reconfirmed the attributes identified in the literature at the theoretical phase and discovered new attributes (see Table 2).

### Physical dimensions

“Ability for daily living” and “symptom management” were derived from the physical dimensions. Specifically, the ability for daily living was mentioned more by older people with dementia than by caregivers. This attribute included maintaining independence in daily living and setting personal goals for daily living ability. Participants felt that living well meant maintaining their independence and continuing their daily lives according to goals that fit their levels.

*“If I do not do what I have been working for all my life, I will get sick. I think I have to go out to the store and see the goods and meet customers…” (Older people 1)*.

Symptom management included cognitive enhancement, behavioral and psychological symptom management, and physical disease and symptom management. Participants underwent rehabilitation training to improve cognitive function and manage behavioral and psychological symptoms accompanied by physical diseases and symptoms for living well.

*“I feel my concentration and memory keep declining… (Omitted) I think I need to know in advance about the decline in concentration and memory, inform my family, and prepare myself.” (Older people 4)*.

*“I was so shocked when my husband (older people with dementia) did not recognize me at first. And then I had a tough time. My husband’s behavior has become strange, and he cannot manage going to the toilet by himself.” (Family 2)*.

### Mental dimensions

The mental dimensions included “psychological health,” “psychological stability,” “maintaining identity,” and “human dignity.” Psychological health included self-efficacy, hope, and willingness. Despite suffering from dementia, participants pursued living well by maintaining a sense of self-efficacy, hope, and the will to live for the future. Notably, while older people with dementia expressed their willingness to live well, caregivers showed negative perceptions toward this concept.

*“Willingness to live well” (Older people 4)*.

*“I never considered how older people with dementia lived well.” (Family 1)*.

*“Well…Is this a realistic concept for these people?” (Nurse 1)*.

Psychological stability included normality and comfort. Participants considered living well in their ordinary lives necessary and wanted a comfortable life and a sense of security in their everyday life.

*“To eat well, get along well with family. Living docilely is the best way to live.” (Family 1)*.

*“The patient must be psychologically stable.” (Nurse 2)*.

Maintaining identity included a sense of self, self-esteem, and a positive view of life. Participants preserved their positive identity and self-esteem and expressed satisfaction and happiness while reflecting on their past lives.

*“I think the way I can live well in the future is to try not to forget to ‘find myself’.” (Older people 4)*.

*“Past experiences still dominate their lives in many cases. (Omitted) Due to the nature of dementia, trying to erase past experiences and memories itself can make the patient worse off, so it’s important to preserve past experiences and memories well and live happily for the rest of your life…” (Nurse 4)*.

Human dignity included meeting basic needs and maintaining a sense of dignity. Participants said that they needed to satisfy their most basic needs related to human life and wanted to maintain dignity throughout their lives.

*“I do not think that just because older people have dementia, their dignity should be denied. In particular, nurses are healthcare professionals who provide care and must be able to protect the dignity of these people to the very end.” (Nurse 1)*.

### Social relationship dimensions

The social relationship dimensions included “maintaining social relations and community connectivity” and “government support.” The first attribute comprised support from the primary caregiver, social engagement, and relationship building. Participants felt that the support of the family primary caregiver significantly impacted their ability to live well. However, some older people with dementia were concerned regarding the primary caregiver’s support load. Participants thought it was necessary to maintain and participate in a participatory and active life, and have community connectivity.

*“I am not good at talking, and it is hard for me to recognize people, but my wife does everything. I think it is good to be with my wife and live happily with her for the rest of my life.” (Older people 2)*.

*“Even though I cannot help my children, it is a parent’s duty not to burden them. I think I am blocking the future of my sons and daughters.” (Older people 3)*.

Government support included facilities for older people with dementia, financial support, and professional workforce support. Notably, families and nurses—rather than older people with dementia—were aware of the importance of receiving support via a social welfare system, such as facilities that can alleviate the financial burden of older people with dementia and caregivers who can provide sufficient specialized services.

*“I think the Ministry of Health and Welfare should provide a lot of support to geriatric hospitals like ours so that older people with dementia can live well, and provide workforce support so that more people can work to ensure the older people with dementia can receive care with dignity.” (Nurse 3)*.

#### Final definition

In the final analytic phase, the attributes for living well for older people with dementia extracted in the theoretical and fieldwork phases were comparatively analyzed and rearranged. Based on this, we derived an integrated final definition.

Among the physical dimensions, “ability for daily living” and “symptom management” were common in the theoretical and fieldwork phases. Among the mental dimensions, “psychological stability” and “psychological health” were repeatedly derived in the two phases. Additionally, while “maintaining identity and growth” was a similarly derived attribute in the theoretical and fieldwork phases, “personal growth” appeared in the theoretical phase. Finally, “human dignity” was confirmed during the fieldwork phase. Among the social relationship dimensions, “maintaining social relations and community connectivity” and “government support” were commonly derived from the theoretical and fieldwork phases.

Based on this, we defined living well for older people with dementia as follows:

The physical dimensions of living well are maintaining the ability to live their life and manage symptoms on a daily basis; the mental dimensions are being psychologically healthy and stable, maintaining identity and human dignity, and facilitating growth; and the social relationship dimensions are maintaining social relations and community connectivity, and ensuring government support (see Table 3).

**Table 3:** Dimensions and attributes of the concept of “living well” for older people with dementia in the final analytic phase

Antecedents	Dimension	Theoretical phase	Fieldwork phase	Final analytic phase	Consequences
• Person-centered approach • Perception of dementia • Shared decision-making	Physical	Ability for daily living	Ability for daily living	Ability for daily living	• Psychological adaptation • Adaptation to disease
		Symptom management	Symptom management	Symptom management	
	Mental	Psychological health	Psychological health	Psychological health	
		Psychological stability	Psychological stability	Psychological stability	
		Maintaining identity and growth	Maintaining identity	Maintaining identity and growth	
		-	Human dignity	Human dignity	
	Social relationship	Maintaining social relations and community connectivity	Maintaining social relations and community connectivity	Maintaining social relations and community connectivity	
		Government support	Government support	Government support	

Thus, for older people with dementia, living well refers to good physical and mental health that responds to changes in daily lifeand positive relationships with people and society.

## Discussion

While developing the concept of living well for older people with dementia, we confirmed most of the attributes identified during the theoretical phase in the fieldwork phase. We subsequently derived eight attributes belonging to the physical, mental, and social relationship dimensions. Among the three dimensions, the mental dimension seemed particularly important as it was mentioned most frequently in the literature. Interestingly, “growth” was added to the conceptualization of living well for older people with dementia. Furthermore, “human dignity” was derived as an additional attribute, reflecting the characteristics observed in the fieldwork. Therefore, in this section, the attributes of the mental dimension are discussed first, followed by those of the physical and social relationship dimensions.

The mental dimensions comprised “psychological health,” “psychological stability,” “maintaining identity and growth,” and “human dignity.” Psychological health was the most frequently mentioned attribute in the literature. Good psychological health can improve self-efficacy, optimism, hope, humor, and resilience [3, 12, 22, 28–31]. Several psychological health factors overlap; however, when combined, they are expected to provide greater resilience in coping with the challenges of living with dementia [29]. In a comprehensive model study of factors related to well-being in people with dementia, psychological characteristics, and health were the only predictors of living well [12]. Considering the social, environmental, and physical factors that support the psychological health of older people with dementia [12], improvements in psychological health can be essential for older people with dementia to live well.

Regarding “psychological stability,” data from the interviews confirmed that for older people with dementia, living well is not a special, positive experience but rather the experience of a normal, comfortable, and stable state. Older people with dementia exhibit a strong willingness to maintain their lives the same as before their diagnosis [31]. Therefore, adequate and appropriate support is needed to ensure they feel psychologically stable and comfortable despite their physical and mental limitations.

The third attribute, “maintaining identity and growth,” was common in both the theoretical and fieldwork phases, while “personal growth” was derived from the literature. By synthesizing 27 qualitative studies, Wolverson et al. [31] confirmed that older people with dementia transcend their condition to maintain their identity and grow individually. The fieldwork phase revealed self-esteem and positive life reviews. However, the attributes of allowing new opportunities for self-understanding, growth, and transcendence suggested in the literature were not identifed during fieldwork. Few studies have examined growth and transcendence experiences in the lives of older people with dementia, and this is a vital new area for further research [31]. Therefore, augmenting the positive identity and self-esteem of older people with dementia and helping them transcend and grow are crucial areas for the clinical and research fields and deserve further research.

The fourth attribute, “human dignity,” was confirmed during fieldwork. The World Health Organization [1] reported that people with dementia are frequently denied their basic rights, freedoms, and choices that others enjoy, and that a treatment environment based on international human rights standards needs to be guaranteed. We also observed that older people with dementia were recognized as needing care. By emphasizing the caregiving burden, efforts were made to prioritize managing their challenging behaviors and cognitive issues. However, this emphasis led to a concurrent decrease in person-centered care.In 1992, Kitwood and Bredinm [44] proposed a person-centered care model that focuses on respecting the people with dementia as a person, which differs from the existing medical model. Further attention is required to promote person-centered care for older people with dementia and maintain their human dignity [16, 33, 37].

The physical dimensions comprised “ability for daily living” and “symptom management.” Regarding the first attribute, “ability for daily living,” research has indicated that even in older people without dementia, independence in daily life can affect their experience of living well [45, 46]. If the ability for daily living in older people with dementia decreases owing to their diagnosis, it can lead to negative consequences, such as reduced social relationships and activities; therefore, interventions to preserve their ability related to daily living are key [47]. This attribute was derived from the particular perspective of older people with dementia, who thought they could continue their daily lives before diagnosis and wanted it to continue but were asked to stop doing so by their families. Studies have suggested that the negative perceptions that older people with dementia experience in their daily life can hinder their ability to live well [3, 26, 33]. Notably, some stereotypes of dementia are so implicit that numerous people do not realize that they are using them [48]. A previous study reported that people with dementia were encouraged to rely on family and friends to perform daily tasks and make decisions rather than implicitly maintaining their own independence [26]. Therefore, it is necessary to reduce the public’s societal judgment, increase their understanding of dementia, and actively support older people with dementia in maintaining their independence by focusing on preserving their abilities. Furthermore, the literature emphasizes the possibility of rehabilitation for older people with dementia and the need for practical interventions considering their individual goals, environment, and risk factors. Therefore, interventions must be developed with an individual problem-solving approach so that older people with dementia can maintain their daily living abilities at the highest possible levels [48].

The second attribute was “symptom management.” The extant literature reports that efforts to ensure that older people with dementia live well should consider various aspects, not just managing dementia symptoms [3]. Notably, extant research has shifted a shift from focusing on the symptoms and defects of older people with dementia to a broader perspective that recognizes their personality and rights, enables optimal functioning, and focuses on participation and inclusion [12]. However, older people with dementia and their families are the ones who face dementia symptoms. If these symptoms worsen, maintaining daily life becomes difficult. Thus, symptom management is an essential primary attribute of the physical dimension.

The social relationship dimensions comprised “maintaining social relations and community connectivity” and “government support.” We will first discuss the first attribute, “maintaining social relations and community connectivity.” In a longitudinal cohort study conducted across England, Scotland, and Wales, 1,547 people with mild-to-moderate dementia were surveyed. Approximately 30.1% and 5.2% reported feeling moderate and severe loneliness, respectively, related to a decline in well-being [49]. Meanwhile, some older people that we surveyed expressed their regret regarding the caregiver’s burden of support. This may be a perception that reflects the characteristics of the Korean family system. In Korea, the number and function of family caregivers are declining owing to nuclear families, an increase in female employment, a weakening of family ties, and a decrease in the number of family members [13, 50]. Consequently, a social care and cooperation system must be created in collaboration with the local community to alleviate the burden on the primary caregiver and strengthen the community’s responsibility. Notably, social relationship support for older people with dementia can help them maintain and promote stable functioning, thus contributing to living well [32]. Therefore, more excellent support is required to ensure that older people with dementia can establish or maintain positive relationships with others and preserve a sense of interconnectedness by continuously participating in social activities.

The second attribute was “government support.” Reportedly, people with dementia or caregivers do not take responsibility for living well, and the government and society need to support people with dementia adequately [3]. In Korea, although the introduction of the long-term care insurance system for older people has promoted the diversification of providers of care services, an insufficient number of facilities are operated by public institutions and their role is insignificant [51]. Therefore, public institutions must expand facilities (e.g., daycare centers) to reflect regional characteristics and needs, ease financial burdens, and create conditions for their use. In addition, a leading and exemplary model is required in which older people with dementia are respected, and appropriate individual care is achieved by fostering and supporting a professional workforce. Furthermore, older people with dementia and their caregivers felt that access to dementia services was complicated and services were provided according to the severity of their condition rather than as a preventive measure [33]. To reduce the long-term burden of dementia in our society, the focus must be on positive concepts about the living, care, and treatment of older people with dementia and promoting their health.

In several studies, living well was perceived as a positive evaluation of quality of life, well-being, and life satisfaction [6, 11, 12, 22, 29]. As each element contains overlapping but distinct elements, scholars argue that integrating them is necessary [11, 12]. Therefore, along with the quality of life, well-being, and life satisfaction, the development of tools for living well for older people with dementia should be implemented by embracing the multidimensional concepts of physical, mental, and social relationships identified in this study.

Our comprehensive conceptualization of living well for older people with dementia is meaningful as it provides essential data for developing tools and effective support and interventions for older people with dementia. Improving their quality of life can contribute to their psychological and long-term adaptation to the disease. By comprehensively considering the attributes, antecedents, and consequences revealed in this study, health and social care professionals can help people with dementia appropriately understand and promote the concept of living well to help them live well.

Using Barcott and Kim’s hybrid model [17], this study has the strength of applying both theoretical and empirical analyses for conceptualizing living well for older people with dementia. However, the in-depth interviews in the field primarily included the viewpoints of care providers and nurses of older people with mild cognitive impairment or mild-to-moderate dementia. Thus, our findings have limited applicability to settings with older people with severe dementia whose limited daily living abilities are severely impaired. Additionally, as the participants were residents of Korea, caution should be exercised while generalizing the results in other socio-cultural contexts.

## Conclusions

This study used theoretical and empirical analyses using a hybrid model to comprehensively identify the attributes of living well for older people with dementia. We found attributes related to the physical, mental, and social relationship dimensions. The respective attributes of these dimensions were “ability for daily living” and “symptom management”; “psychological health,” “psychological stability,” “maintaining identity and growth,” and “human dignity”; and “maintaining social relations and community connectivity” and “government support,” respectively. Crucially, our results are meaningful as they provide basic data for understanding the concept and developing tools and interventions incorporating the physical, mental, and social relationship dimensions related to living well based on the perspectives of older people with dementia, their families, and nurses. A vital area is promoting and improving living conditions, which is an important concept in implementing a person-centered approach for older people with dementia. Furthermore, follow-up studies should be conducted to improve the quality of the clinical field by developing practical interventions for older people with dementia.

## Data Availability

All data generated or analyzed during this study are included in this manuscript.

## Abbreviations

USD: United States dollar
KRW: Korean Won
